# Upconverting Nanoparticles Functionalized with Protein–Gold Nanoclusters and Chlorin e6 for Near-Infrared-Activated Photodynamic Therapy

**DOI:** 10.3390/nano16070417

**Published:** 2026-03-30

**Authors:** Vilius Poderys, Greta Butkiene, Dziugas Jurgutis, Aleja Marija Daugelaite, Egle Ezerskyte, Vaidas Klimkevicius, Vitalijus Karabanovas

**Affiliations:** 1 Biomedical Physics Laboratory, National Cancer Institute, P. Baublio str. 3b, LT-08406 Vilnius, Lithuania; vilius.poderys@nvi.lt (V.P.); greta.jarockyte@nvi.lt (G.B.); dziugas.jurgutis@nvi.lt (D.J.); aleja.daugelaite@nvi.lt (A.M.D.); egle.ezerskyte@chgf.stud.vu.lt (E.E.); vaidas.klimkevicius@chf.vu.lt (V.K.); 2 Institute of Chemistry, Faculty of Chemistry and Geosciences, Vilnius University, Naugarduko str. 24, LT-03225 Vilnius, Lithuania; 3 Department of Chemistry and Bioengineering, Vilnius Gediminas Technical University, Sauletekio av. 11, LT-10221 Vilnius, Lithuania

**Keywords:** protein corona, cancer, PDT, reactive oxygen species, energy transfer

## Abstract

Current efforts to improve photodynamic therapy focus on nanomaterials that integrate deep tissue imaging with efficient reactive oxygen species generation. Gold nanoclusters (Au NCs) are promising alternatives to conventional photosensitizers due to their effective ROS production and enhanced biocompatibility when stabilized by a protein corona. However, both photosensitizers and Au NCs are typically activated by ultraviolet or visible light, which cannot penetrate deeper into tissues and is limited to superficial applications. Here, we report a near-infrared (NIR)-activated photodynamic nanoplatform based on core–shell upconverting nanoparticles (UCNPs; NaGdF_4_:Yb^3+^,Er^3+^@NaGdF_4_:Yb^3+^,Nd^3+^), functionalized with a protein corona containing bovine serum albumin-stabilized Au NCs (BSA–Au NCs) and photosensitizer chlorin e6 (Ce6). Spectroscopic data confirmed the formation of the UCNP-BSA–Au-Ce6 nanoplatform and demonstrated 32% energy transfer efficiency from UCNPs to Ce6, resulting in efficient reactive oxygen species generation under 808 nm irradiation. Cellular experiments confirmed the effective internalization and optimal biocompatibility of the nanoplatform in human breast cancer and healthy cells. Upon irradiation at 808 nm, the nanoplatform significantly reduced the viability of MDA-MB-231 cancer cells. These findings indicate that the UCNP-BSA–Au-Ce6 nanoplatform couples NIR activation with enhanced singlet oxygen production, providing a multifunctional platform for deep tissue imaging and NIR-activated photodynamic therapy.

## 1. Introduction

Photodynamic therapy (PDT) is an established cancer treatment method that employs light-activated compounds, known as photosensitizers (PSs), which produce reactive oxygen species (ROS) upon exposure to light [1]. The generated ROS cause cellular damage that could lead to the death of cancer cells during PDT. However, most PSs have several disadvantages, such as nonspecific accumulation in healthy tissues, low water solubility, low photostability, and cytotoxicity. Although newer PSs have been developed to mitigate many of these limitations, achieving the selective accumulation of PSs in tumor tissues remains a major challenge [2,3].

The rapid development of nanotechnology in the past few decades has also offered innovative solutions for cancer diagnosis and treatment [3,4]. There have been many efforts to develop nanomaterials that produce ROS upon light irradiation and can be applied for PDT. Among the wide range of nanomaterials under investigation, gold nanoclusters (Au NCs) have attracted significant attention as promising agents for cancer therapy. Currently, Au NCs are among the few nanomaterials capable of competing with PSs in terms of ROS generation [5,6]. Protein-stabilized Au NCs are particularly attractive due to their environmentally friendly synthesis and biocompatibility [7]. A variety of proteins can be used for Au NC synthesis [8,9], including bovine serum albumin (BSA) [8] and human serum albumin (HSA) [10,11]. BSA and HSA are homologous proteins with similar functions, such as the transport of small molecules and the maintenance of osmotic balance, but they differ in specific properties, including hydrophobicity, stability, and crystallization behavior [12]. The lower cost and availability of BSA have led to extensive research on BSA-stabilized gold nanoclusters (BSA–Au NCs), resulting in a better understanding of these systems compared with other protein-stabilized Au NCs. Previously, our group demonstrated the biocompatibility of BSA–Au NCs and their accumulation in cancer cells [13], as well as ROS generation and the destruction of cancer cells upon visible (VIS) light irradiation in vitro [6]. In addition, we showed that BSA–Au NCs are eliminated through the urinary system in vivo [14]. Furthermore, we demonstrated that Au NCs can be synthesized in human blood plasma, and the obtained Au NCs exhibit properties similar to those of BSA–Au NCs. This suggests that personalized Au NCs could potentially be applied to patients in the future [15].

BSA–Au NCs could be a promising agent for PDT; however, their activation with ultraviolet (UV) or visible light limits their application to superficial tissues. In contrast, near-infrared (NIR) light can penetrate deeper into biological tissues but lacks sufficient energy to excite BSA–Au NCs via single-photon absorption directly. BSA–Au NCs can be excited by NIR light via a two-photon absorption process [16]; however, this approach requires expensive ultrafast lasers and high-speed scanning systems. Additionally, it is often difficult to avoid thermal effects because of the extremely high laser power densities required for nonlinear excitation. To address these limitations, upconverting nanoparticles (UCNPs) offer a promising solution [17,18,19]. The upconverted emission from excited UCNPs can be transferred to BSA–Au NCs, promoting their excitation and subsequent generation of ROS required for PDT. This mechanism enables deeper tissue penetration while maintaining the ability to activate BSA–Au NCs. A key advantage of such a nanoplatform is that UCNP excitation can be achieved via steady-state NIR lasers at relatively low power densities compared to those required for two-photon excitation.

Once nanoparticles are exposed to protein-rich media, the formation of a protein corona (sometimes referred to as a biomolecular corona [20]) around the nanoparticle surface begins. The protein corona can alter nanoparticle properties and determine their interaction with the biological environment [21]. The surface modification of nanoparticles and composition of the surrounding media are the main factors determining the formation of the protein corona [22,23]. Despite extensive research, current knowledge of the protein corona remains insufficient to accurately predict the behavior of nanoparticles after their administration to an organism [24]. Two main strategies are commonly used to minimize undesirable effects associated with protein corona formation: preventing protein adsorption or controlling it. The first strategy involves modifying the nanoparticle surface to reduce protein binding. Various approaches have been proposed, although polyethylene glycol functionalization is the most widely used [25]. However, the complete elimination of protein adsorption on nanoparticle surfaces remains challenging [24]. Alternatively, a protein corona can be intentionally formed under controlled conditions using selected proteins before in vitro or in vivo applications. For example, the controlled formation of a BSA protein corona on UCNPs improves their colloidal and physiological stability and can serve as a delivery platform for loading various therapeutic agents [26,27]. To the best of our knowledge, no nanoplatforms combining UCNPs with BSA–Au NCs have been reported to date.

In this study, BSA-Au NCs were used to form a protein corona around core–shell UCNPs (NaGdF_4_:Yb^3+^,Er^3+^@NaGdF_4_:Yb^3+^,Nd^3+^). This system was designed to combine the deep tissue penetration of NIR light with the ROS-generating capabilities of the nanoclusters. The ability of the UCNP-BSA–Au nanoplatform to generate ROS was evaluated using fluorescent ROS sensor dihydrorhodamine 123 (DHR123). However, ROS generation by the UCNP-BSA–Au nanoplatform under NIR irradiation was insufficient to achieve effective photodynamic activity. To enhance ROS generation, a complex of BSA–Au NCs and photosensitizer chlorin e6 (Ce6) was prepared before its assembly as a protein corona on UCNPs. Upon NIR irradiation, the UCNP-BSA–Au-Ce6 nanoplatform exhibited significantly higher ROS generation and improved performance compared to the nanoplatform without Ce6. Additionally, the cellular accumulation of nanoplatforms was evaluated in breast cancer and healthy cells. The results showed that the nanoplatforms were internalized into cells and did not exhibit cytotoxicity in the absence of NIR irradiation. In contrast, treatment with the UCNP-BSA–Au-Ce6 nanoplatform followed by irradiation with an 808 nm laser significantly reduced cancer cells’ viability, indicating its potential as a phototherapeutic agent for NIR-activated photodynamic therapy.

## 2. Materials and Methods

In this study, NaGdF_4_:Yb^3+^ (18%), Er^3+^ (2%)@NaGdF_4_:Yb^3+^ (5%), Nd^3+^ (40%) core–shell UCNPs were used. These core–shell UCNPs were synthesized via a two-step thermal coprecipitation route following a procedure previously reported by our research group [28]. BSA–Au NCs were synthesized according to the protocol described by Xie et al. [8], with minor modifications. Oleate ligands were removed from UCNP surfaces by treatment under acidic conditions, resulting in water-dispersible nanoparticles. The ligand-free UCNPs were then immersed in a solution containing BSA–Au NCs or BSA–Au NCs with the photosensitizer Ce6 to form a protein corona. The generation of ROS and singlet oxygen by the UCNP-BSA–Au and UCNP-BSA–Au–Ce6 nanoplatforms under 808 nm laser irradiation was evaluated using the fluorescent probes DHR123 (ROS) and SOSG (singlet oxygen). Detailed methodologies describing the synthesis and characterization of UCNPs and BSA–Au NCs, as well as the procedures for the preparation of the UCNP-BSA–Au and UCNP-BSA–Au-Ce6 nanoplatforms and the assessments of their physicochemical properties, are provided in the Supplementary Materials.

Cellular experiments included conditions for the cultivation of breast cancer and healthy breast epithelial cells. The cellular accumulation of UCNPs in these cells was visualized by confocal laser scanning microscopy. The PDT effect was evaluated in vitro using MDA-MB-231 cancer cells. Cell viability after incubation with nanoparticles and irradiation with an 808 nm laser was assessed using fluorescent viability dyes: Calcein-AM (Invitrogen) for live cell staining and propidium iodide (PI; Carl ROTH, Karlsruhe, Germany) for dead cell staining. Additionally, cell metabolic activity was measured using the Cell Counting Kit-8 (CCK-8; Vazyme, Nanjing, China). Detailed descriptions of all cellular experimental procedures are provided in the Supplementary Materials.

## 3. Results and Discussion

### 3.1. Formation of UCNP-BSA–Au Nanoplatform and ROS Generation

**Figure 1:**
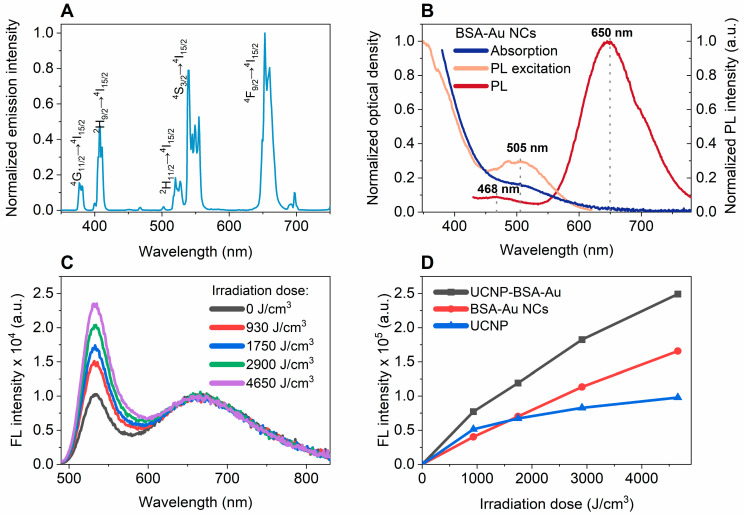
(**A**) Emission spectrum of NaGdF_4_:Yb^3+^,Er^3+^@NaGdF_4_:Yb^3+^,Nd^3+^ core–shell UCNPs in UV/VIS region (λ_ex_ = 808 nm). (**B**) Spectroscopic analysis of BSA–Au NCs: absorption (blue), PL excitation (light orange, λ_em_ = 650 nm), and PL (red, λ_ex_ = 405 nm). (**C**) Evolution of Rh123 fluorescence spectrum (λ_ex_ = 480 nm) during irradiation of formed UCNP-BSA–Au nanoplatform with 808 nm laser. (**D**) Changes in integrated Rh123 fluorescence (515–550 nm, λ_ex_ = 480 nm) during 808 nm laser irradiation for three samples: UCNP-BSA–Au nanoplatform (black), BSA–Au NCs (red), and UCNPs (blue).

Firstly, NaGdF_4_:Yb^3+^,Er^3+^@NaGdF_4_:Yb^3+^,Nd^3+^ core–shell UCNPs were freshly synthesized and spectroscopically evaluated. Figure 1A represents the upconversion emission spectrum of nanoparticles dispersed in aqueous media (DI water, pH = 5.5). Upon excitation with a continuous-wave 808 nm laser, the emission spectra exhibit five major bands in the UV-Vis region, characteristic of Er^3+^ electronic transitions. The observed emission peaks correspond to the following transitions: ^4^G_11/2_→^4^I_15/2_ (ca. 377 nm), ^2^H_9/2_→^4^I_15/2_ (ca. 407 nm), ^2^H_11/2_→^4^I_15/2_ (ca. 519 nm), ^4^S_3/2_→^4^I_15/2_ (ca. 539 nm), and ^4^F_9/2_→^4^I_15/2_ (ca. 653 nm). The upconversion emission process in NaGdF_4_:Yb^3+^,Er^3+^@NaGdF_4_:Yb^3+^,Nd^3+^ core–shell UCNPs is initiated by the absorption of 808 nm photons by Nd^3+^ located in the outer shell layer. Photons from the excited states of Nd^3+^ (^4^F_5/2,_^4^F_3/2_) are transferred to neighboring Yb^3+^ (^2^F_5/2_). The excited Yb^3+^ ions serve as intermediate sensitizers and can effectively transfer energy and populate the higher-lying energy states of Er^3+^ located in the cores of UCNPs. Radiative relaxation from the excited levels of Er^3+^ to the ground state (^4^I_15/2_) results in characteristic emission bands in the UV–blue, green, and red regions of the upconversion emission spectrum. A detailed energy level scheme and the corresponding optical transitions have been reported in our previous publication [28].

Secondly, BSA-stabilized gold nanoclusters were synthesized and spectroscopically evaluated. The absorption, photoluminescence (PL) excitation, and PL spectra of the BSA–Au NCs are presented in Figure 1B. The synthesized BSA–Au NCs exhibit a pronounced PL band in the red spectral region with a maximum at approximately 650 nm, accompanied by a weaker emission band centered at around 468 nm. The PL excitation spectrum shows a distinct band with a maximum at 505 nm. The absorption spectrum shows no distinct peaks; however, a shoulder is observed around 505 nm, coinciding with the PL excitation maximum. Overall, absorption increases toward shorter wavelengths, which is characteristic of ultrasmall Au NCs lacking a surface plasmon resonance band.

The presence of a PL maximum at 650 nm confirms the successful formation of BSA–Au NCs. The number of gold atoms in the cluster can be estimated using the following relation [29]:

(1) $$N={\left(\frac{e\lambda_{\max}E_{F}}{hc}\right)}^{3},$$

where N is the number of gold atoms in the nanocluster, $\lambda_{\max}$ is the maximum of the fluorescence emission band, $E_{F}$ is the Fermi energy of gold expressed in eV, e is the elementary charge, h is Planck’s constant, and c is the speed of light.

Using this equation, the synthesized BSA–Au NCs were estimated to contain approximately 24 gold atoms per nanocluster. This value is in good agreement with literature reports, particularly the study by Xie et al. (2009), which describes the formation of Au_(25)_ clusters under similar synthesis conditions [8].

The efficient formation of a BSA–Au NC protein corona around UCNPs, as well as subsequent energy transfer processes, requires close interparticle interactions, primarily driven by surface chemistry or electrostatic forces. Therefore, the surface charge characteristics of both components were evaluated by measuring their zeta potential as a function of pH. The zeta potentials of BSA–Au NCs and UCNPs were determined over a pH range of 3–11 (Supplementary Material Figure S1). Across the pH range of 5–10, the BSA–Au NCs exhibited a negative zeta potential, indicating an overall negatively charged surface. The isoelectric point (IEP) was identified at pH 4.2, where the net surface charge approaches zero, leading to reduced colloidal stability due to aggregation. At pH values below 4.2, the zeta potential becomes positive. The measured IEP is in close agreement with reported values for native BSA (pI ≈ 4.5) [30], indicating that the incorporation of Au NCs within the protein matrix does not significantly perturb the intrinsic electrochemical properties of BSA. This observation suggests the preservation of the protein structure and supports the expected biocompatibility of the synthesized BSA–Au NCs.

The pH-dependent zeta potential profile of UCNPs (Supplementary Material Figure S1) reveals an isoelectric point at significantly higher pH compared to BSA–Au NCs, identified at pH 8.5. At alkaline pH (pH > 9), UCNPs exhibit a negative surface charge, whereas at lower pH values, the surface becomes positively charged.

During the preparation of the UCNP-BSA–Au nanoplatform, the UCNP solution was adjusted to pH 5.5, at which the zeta potential was approximately +44 mV, indicating a strongly positively charged surface. In contrast, the BSA–Au NCs dispersed at pH 7.4 exhibited a negative zeta potential of approximately −22 mV. Upon mixing, the resulting dispersion had an intermediate pH of 5.5–7.4. Within this pH range, UCNPs remain positively charged, while BSA–Au NCs remain negatively charged, establishing favorable electrostatic conditions for adsorption. The oppositely charged surfaces promote attractive interactions, leading to the spontaneous formation of a protein-based corona of BSA–Au NCs around the UCNPs.

To experimentally verify this electrostatically driven association, centrifugation-based separation experiments were performed. Dispersions containing UCNPs, BSA–Au NCs, or their mixtures were centrifuged at 14,500 RPM, and PL spectra were recorded for the initial sample (control) and for the supernatant and the redispersed pellet after centrifugation. BSA–Au NCs alone did not sediment under the applied centrifugal force, and no pellet was formed, indicating the homogeneous distribution of nanoclusters throughout the solution. In contrast, UCNPs alone exhibited negligible emission in the supernatant and strong UC emission in the redispersed pellet, confirming efficient sedimentation due to their larger size and higher mass.

For the UCNP-BSA–Au nanoplatform, a pronounced redistribution of BSA–Au NC PL was observed after centrifugation (Supplementary Material Figure S2). The PL intensity associated with BSA–Au NCs was significantly reduced in the supernatant and correspondingly increased in the redispersed pellet. This behavior observed in the UCNP and BSA–Au NC mixture indicates the co-sedimentation of BSA–Au NCs with UCNPs and therefore confirms the adsorption of the nanoclusters onto the UCNP surface. Additionally, the emission measurements of the centrifuged UCNP–BSA–Au nanoplatform samples revealed a negligible signal in the supernatant fraction, further supporting the efficient sedimentation of UCNP-containing nanoplatforms. Collectively, these results demonstrate that electrostatic attraction between positively charged UCNPs and negatively charged BSA–Au NCs drives spontaneous surface decoration and the formation of a stable UCNP-BSA–Au nanoplatform. Although the qualitative formation of the nanoplatform is clearly evidenced, the exact surface coverage and quantitative loading of BSA–Au NCs on UCNPs cannot be directly determined from centrifugation experiments alone.

It has been previously reported that BSA–Au NCs generate ROS upon excitation with blue light [6]. Because the PL excitation spectrum of BSA–Au NCs overlaps with the emission bands of UCNPs, it can be anticipated that UCNPs may act as energy donors for the nanoclusters via an energy transfer process (Supplementary Material Figure S3A). Such a mechanism could potentially enable ROS generation under NIR excitation via the upconversion-mediated activation of UCNP-BSA–Au. To test this hypothesis, ROS generation in the UCNP-BSA–Au nanoplatform was investigated using the ROS-sensitive probe DHR123, which is oxidized by ROS to the fluorescent dye rhodamine 123. The changes in the fluorescence spectra of the DHR123 probe during the irradiation of the UCNP-BSA–Au nanoplatform with an 808 nm laser are presented in Figure 1C. The NIR irradiation of the UCNP-BSA–Au nanoplatform leads to a gradual increase in the fluorescence intensity of the Rh123 band centered at ~525 nm (Figure 1D). This increase indicates that ROS are generated in the solution when the complex is excited with 808 nm radiation. However, the magnitude of fluorescence growth is relatively small. Only a minor difference is observed when comparing the signal increase in the UCNP-BSA–Au nanoplatform sample with that in the control samples containing either UCNPs or BSA–Au NCs alone (Figure 1D). These results suggest that although ROS generation can be detected under 808 nm excitation, the efficiency of ROS production in the UCNP-BSA–Au nanoplatform is low. The weak increase in the DHR123 fluorescence signal indicates that the indirect excitation of BSA–Au NCs mediated by UCNP emission does not lead to efficient ROS generation under the applied experimental conditions.

To evaluate the occurrence of energy transfer from UCNPs to BSA–Au NCs, the PL decay kinetics of UCNP emission were measured for both ligand-free UCNPs and UCNPs surrounded by a BSA–Au NC corona. The decay kinetics were analyzed for the characteristic UCNP emission bands at 407 nm, 549 nm, and 653 nm (Supplementary Material Figure S4). The decay curves were fitted to a biexponential model, which is commonly used to describe the population dynamics and relaxation processes of excited states in UCNPs. The first exponential component exhibits a negative amplitude, indicating a rise component that reflects the population build-up dynamics of the emitting level rather than its relaxation. In UCNP systems, this component is typically associated with excited-state population processes governed by the energy transfer upconversion (ETU) mechanism. The second exponential component corresponds to the decay of the populated excited state. It therefore reflects the relaxation dynamics of the emitting level, including radiative emission, non-radiative processes, or energy transfer. The shortening of the lifetime of this second decay component (*τ*_2_) is an indication of the presence of an additional relaxation pathway associated with energy transfer from the excited UCNP states. The decay parameter *τ*_2_ determined for UCNPs was 74, 119, and 213 µs, for emission bands at approximately 407, 549, and 653 nm, respectively. For UCNPs surrounded by the BSA–Au NC corona, the corresponding *τ*_2_ decay times were 76 µs (407 nm), 126 µs (549 nm), and 213 µs (653 nm). Non-radiative energy transfer from UCNPs to BSA–Au NCs would be expected to shorten the *τ*_2_ decay lifetime. However, the decay times *τ*_2_ measured for ligand-free UCNPs and for the UCNP-BSA–Au nanoplatform are virtually identical within experimental uncertainty. This indicates that the presence of BSA–Au NCs does not introduce an additional non-radiative relaxation pathway for the excited states of UCNP, suggesting that non-radiative energy transfer from UCNPs to BSA–Au NCs is either absent or extremely inefficient. These observations imply that the excitation of the BSA–Au NCs in the nanoplatform most likely occurs via radiative processes, namely through the reabsorption of UCNP emission by the BSA–Au NCs. Such mechanisms are significantly less efficient than non-radiative mechanisms such as Förster resonance energy transfer (FRET). Although partial spectral overlap between UCNPs’ emission and their absorption of BSA–Au NCs enables the indirect excitation of the nanoclusters, this process leads to only weak activation of the nanoclusters and, consequently, inefficient ROS generation in the UCNP–BSA–Au nanoplatform under NIR (808 nm) excitation.

### 3.2. UCNP-BSA-Au-Ce6 Nanoplatform Formation, Investigation of Energy Transfer and Singlet Oxygen Generation

Since the UCNPs with the BSA–Au NC corona did not exhibit efficient ROS generation under NIR excitation, an additional strategy was explored to enhance the photodynamic activity of the system. For this purpose, Ce6, a clinically approved PS that generates singlet oxygen, was incorporated into the UCNP-BSA–Au NC nanoplatform. Ce6 has been widely used in PDT and is known to interact with BSA, which can facilitate its binding to nanoparticles with a protein corona [31].

**Figure 2:**
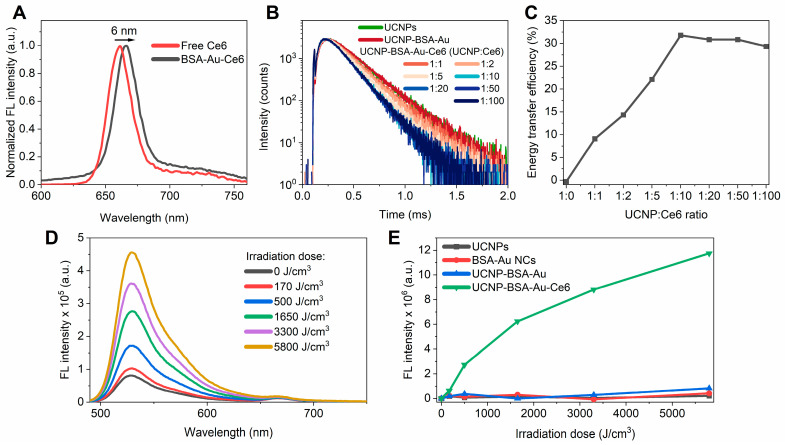
(**A**) Fluorescence spectra of free Ce6 and Ce6 bound to UCNP-BSA-Au nanoplatform. Shift in fluorescence band confirms interaction between Ce6 and BSA (λ_ex_ = 405 nm). (**B**) UCNP emission decay kinetics of 653 nm emission band in different samples: UCNPs, UCNP-BSA–Au nanoplatform, and UCNP-BSA–Au-Ce6 with various UCNP:Ce6 ratios. (**C**) Energy transfer efficiency from UCNP band at 653 nm to Ce6 as function of UCNP:Ce6 ratio. (**D**) Evolution of SOSG-EP fluorescence spectrum (λ_ex_ = 480 nm) during irradiation of UCNP-BSA–Au-Ce6 nanoplatform with 808 nm laser. (**E**) Changes in integrated SOSG-EP fluorescence (515–550 nm, λ_ex_ = 480 nm) during 808 nm laser irradiation for four samples: UCNPs (black), BSA–Au NCs (red), UCNP-BSA–Au nanoplatform (blue), and UCNP-BSA–Au-Ce6 (green).

The fluorescence spectra (λ_ex_ = 405 nm) of free Ce6 in water and in an aqueous solution containing BSA–Au NCs are presented in Figure 2A. The spectra reveal a clear change in the emission characteristics of Ce6 upon the addition of BSA–Au NCs. The fluorescence maximum of free Ce6 in aqueous solution is observed at approximately 661 nm. However, after the addition of Ce6 to the UCNP-BSA–Au nanoplatform, a red shift in the fluorescence band was detected, with the Ce6 fluorescence band maximum shifting to 667 nm at a BSA–Au NC:Ce6 ratio of 1:1. Such a bathochromic shift is consistent with literature reports describing the interaction of Ce6 with BSA molecules [31] and indicates the binding of Ce6 to the protein component of the corona. This behavior is attributed to the incorporation of Ce6 into the hydrophobic binding pockets of the BSA matrix via non-covalent interactions, which alters the local microenvironment of the fluorophore. As the Ce6 concentration was gradually increased, the fluorescence maximum shifted back toward shorter wavelengths (data not presented), approaching the position characteristic of free Ce6. This behavior indicates the saturation of the available binding sites within the protein corona and the increasing contribution of unbound or weakly associated Ce6 species in solution.

The interaction between Ce6 and the protein corona should create favorable conditions for energy transfer from UCNPs to Ce6. The emission band of UCNPs at 407 and 653 nm significantly overlaps with the absorption spectrum of Ce6 (Supplementary Material Figure S3B), suggesting that energy transfer from the UCNP blue and red emission bands to Ce6 is feasible. To evaluate this possibility, the PL decay kinetics of the UCNPs’ emission bands were measured as the Ce6 concentration was gradually increased (Supplementary Material Figure S4). The decay kinetics of the blue (407 nm) and green (549 nm) UCNP emission bands showed almost no change with increasing Ce6 concentration. However, a slight shortening of the lifetime was observed for the 407 nm band, suggesting a minor contribution of energy transfer from this UCNP energy state to Ce6 (Supplementary Material Figure S4A,B). In contrast, a pronounced shortening of the lifetime of the red UCNP emission band at 653 nm was observed as the Ce6 concentration increased (Figure 2B). The *τ*_2_ lifetime of this emission band decreased progressively from 213.2 µs to 145 µs until a molar UCNP:Ce6 ratio of approximately 1:10 was reached (Supplementary Material Table S1). Further increases in Ce6 concentration did not produce additional changes in the decay time. This saturation behavior indicates that only a limited number of Ce6 molecules are located within the effective energy transfer distance from the UCNP surface. The observed plateau at the UCNP:Ce6 molar ratio of approximately 1:10 suggests that up to ten Ce6 molecules can be positioned in close proximity to each nanoparticle. Considering that Ce6 interacts with BSA with an approximately 1:1 binding stechiometry [31], this result allows for the estimation of the number of BSA–Au NCs forming the protein corona around each UCNP. The obtained value indicates that the corona surrounding a single UCNP comprises approximately 10 BSA–Au NCs, consistent with the expected packing density based on their reported hydrodynamic size.

The efficiency of energy transfer from the donor to the acceptor can be calculated from the change in the excited-state relaxation lifetime [32]:

(2) $$\eta =\left(1-\frac{\tau_{UCNP-BSA-Au-Ce6}}{\tau_{UCNP}}\right),$$

where *η* is energy transfer efficiency, *τ_UCNP-BSA-Au-Ce_*_6_ is the *τ*_2_ PL emission decay time of the UCNP-BSA–Au-Ce6 nanoplatform, and *τ_UCNP_* is the *τ*_2_ PL emission decay time of ligand-free UCNPs.

The energy transfer efficiencies for various UCNP:Ce6 ratios are presented in Table 1.

Energy transfer efficiency from the blue UCNP emission band at 407 nm was negligible. In contrast, energy transfer from the red UCNP emission band at 653 nm increased with the UCNP:Ce6 ratio and reached a maximum of approximately 32% at a UCNP:Ce6 ratio of 1:10. These results show that non-radiative energy transfer occurs from the red UCNP emission band to Ce6 molecules bound within the BSA–Au NC corona formed on UCNPs. Since Ce6 is an efficient singlet oxygen generator, this energy transfer pathway suggests that this complex, in which Ce6 is incorporated into the UCNP-BSA–Au nanoplatform corona, can function as an effective photosensitizing system. Consequently, the NIR excitation of UCNPs can activate Ce6 via upconversion-mediated energy transfer, enabling singlet oxygen generation and potentially enhancing the photodynamic therapeutic performance of the system. The production of singlet oxygen in the prepared UCNP-BSA–Au–Ce6 nanoplatform was evaluated using the Singlet Oxygen Sensor Green (SOSG) probe. SOSG reacts with singlet oxygen to form a fluorescent endoperoxide product (SOSG-EP). The formation of SOSG-EP was monitored by measuring the fluorescence intensity of its emission band (λ_max_ = 529 nm). Changes in SOSG-EP fluorescence during the irradiation of the samples with an 808 nm laser are shown in Figure 2D,E.

Only a minor increase in SOSG-EP fluorescence was observed upon the irradiation of UCNPs, BSA–Au NCs, and the UCNP-BSA–Au nanoplatform, indicating that these systems do not produce detectable amounts of singlet oxygen under the applied conditions. In contrast, a pronounced increase in SOSG-EP fluorescence intensity was detected in the UCNP-BSA–Au–Ce6 nanoplatform under identical irradiation conditions.

The significant increase in SOSG fluorescence demonstrates that singlet oxygen is generated only when Ce6 molecules are incorporated into the protein corona surrounding the UCNPs. The observed effect is consistent with the previously identified energy transfer pathway from the red UCNP emission band to Ce6, which activates the photosensitizer and triggers singlet oxygen production.

These results indicate that the UCNP-BSA–Au-Ce6 nanoplatform represents a promising system for NIR-activated PDT. The possibility of excitation with NIR radiation enables PDT at greater tissue depths and expands the potential of such nanostructured theranostic platforms combining PL imaging and PDT.

### 3.3. Accumulation in Cells

**Figure 3:**
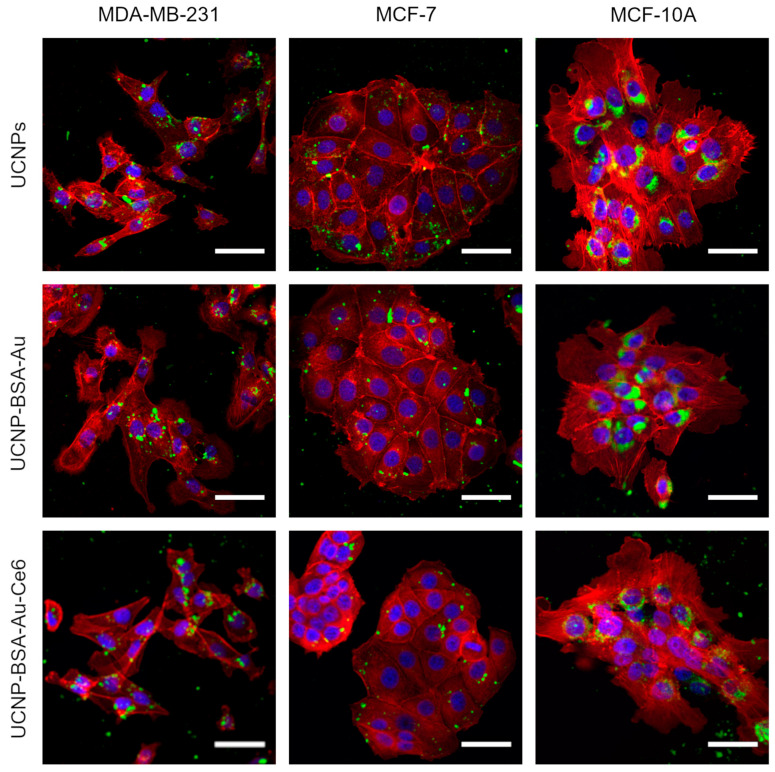
Confocal microscopy images of MDA-MB-231, MCF-7, and MCF-10A cells after incubation with UCNPs and UCNP-BSA–Au and UCNP-BSA–Au-Ce6 nanoplatforms. UCNP emission is shown in green (λ_ex_ = 808 nm), F-actin stained with CF594 dye is shown in red (λ_ex_ = 543 nm), and nuclei stained with Hoechst are shown in blue (λ_ex_ = 404 nm). Scale bars: 50 μm.

Before any biological application of nanoparticles, it is essential to investigate whether they can be internalized into cells. For biological testing, we used the breast cancer cell lines MDA-MB-231 and MCF-7, as well as the MCF-10A cell line, which represents healthy breast epithelial cells. Confocal microscopy analysis revealed that UCNPs and the UCNP-BSA–Au and UCNP-BSA–Au-Ce6 nanoplatforms accumulated in MDA-MB-231, MCF-7, and MCF-10A cells (Figure 3). UCNP emission was detected in cluster- or vesicle-like structures, indicating the endocytic uptake of the nanoparticles [22]. Visually, there was no difference in accumulation between ligand-free UCNPs and the protein-coated nanoplatforms (UCNP-BSA–Au and UCNP-BSA–Au-Ce6). The exposure of nanoparticles to protein-rich media leads to the formation of a protein corona, which alters cellular internalization pathways [22]. In this case, both the UCNP-BSA–Au and UCNP-BSA–Au-Ce6 nanoplatforms had already formed a hard protein corona before introduction into the cell culture medium; therefore, only a soft corona could form subsequently. It is also likely that the ligand-free UCNPs were covered by a protein corona primarily composed of BSA, the most abundant protein in fetal bovine serum [22].

Additionally, UCNPs were coated with BSA conjugated to Alexa555 (BSA-Alexa555) to evaluate whether the BSA coating remains attached to the UCNPs after internalization in cells. The UCNP-BSA-Alexa555 nanoplatform was prepared using the same method as that used for the UCNP-BSA–Au nanoplatform, under identical incubation conditions. During confocal microscopy imaging, the UCNPs’ emission and Alexa555 fluorescence overlapped within the same intracellular regions (Supplementary Material Figure S5). The overlapping signals indicate that the UCNP-BSA-Alexa555 nanoplatform remains stable after internalization. Therefore, the UCNP-BSA–Au and UCNP-BSA–Au-Ce6 nanoplatforms are also expected to remain conjugated within cells.

After incubation with UCNPs, UCNP-BSA–Au, and UCNP-BSA–Au-Ce6, the cells retained their morphology, indicating that the investigated nanoparticles are biocompatible. These findings were further confirmed by the lactate dehydrogenase release assay (Supplementary Material Figure S6).

### 3.4. Photodynamic Effect in Cells

**Figure 4:**
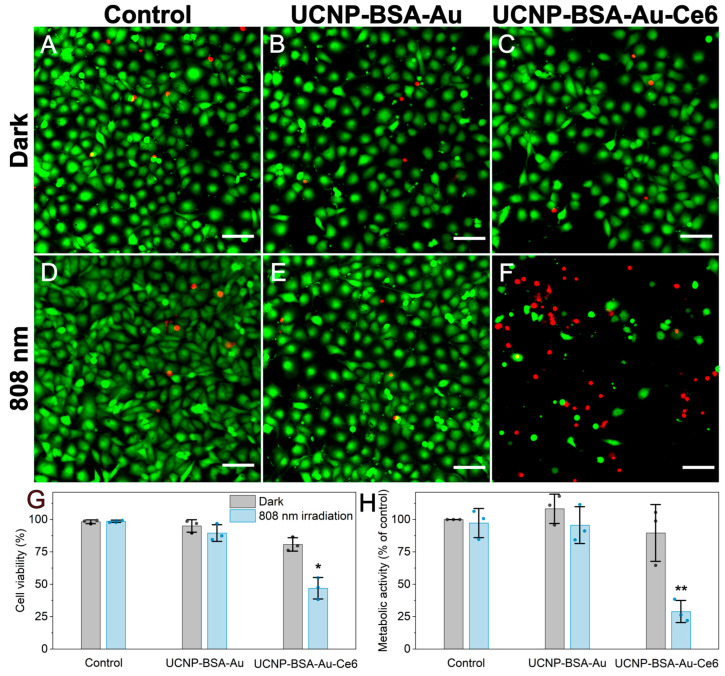
Evaluation of nanoplatform biocompatibility and photodynamic effect in MDA-MB-231 cells. (**A**–**F**) Confocal fluorescence images of MDA-MB-231 cells stained with Calcein-AM (green colour, live cells, λ_ex_ = 488 nm) and propidium iodide (red colour, dead cell nuclei, λ_ex_ = 543 nm) after treatment with UCNP-BSA–Au or UCNP-BSA–Au-Ce6 nanoplatform and irradiation with 808 nm laser. Scale bars: 100 μm. (**G**,**H**) Quantitative analysis of cell viability and metabolic activity based on percentage of live cells among total stained cells, determined from Calcein-AM and propidium iodide images using ImageJ 1.8.0_172 AutoCount macro [41] and by CCK-8 assay, respectively. Data points represent mean of at least three technical replicates per experiment (*n* = 3 independent experiments). Bars indicate group means ± standard deviation. Statistical significance between irradiated control and each treatment group was evaluated using two-sided Welch’s *t*-test with Bonferroni correction for multiple comparisons. Non-irradiated control was excluded from statistical comparisons in CCK-8 assay. Asterisks indicate significant differences from irradiated control: * *p* < 0.05, ** *p* < 0.01.

Having determined the accumulation of nanoplatforms across cell lines, we selected the MDA-MB-231 cell line to evaluate the in vitro photodynamic efficacy of the UCNP-BSA–Au and UCNP-BSA–Au-Ce6 nanoplatforms. Cells were incubated with 0.1 mg/mL of the UCNP-BSA–Au or UCNP-BSA–Au-Ce6 nanoplatform for 24 h and then irradiated with an 808 nm laser. For irradiation, we constructed a custom automated setup based on a modified 3D printer equipped with a microplate holder and an 808 nm diode laser. The system scanned the laser beam across the surface of each well in a line-scanning pattern with 0.5 mm spacing between parallel lines, ensuring the uniform and reproducible irradiation of the entire well area. Importantly, laser irradiation alone had no detectable effect on cell viability. Irradiated control cells retained normal morphology (Figure 4A,D), exhibited a high fraction of Calcein-AM-positive cells (Figure 4D,G), and showed no significant change in metabolic activity (Figure 4H). These findings confirm that the applied NIR irradiation was harmless. The use of 808 nm excitation is advantageous for PDT, as NIR light penetrates deeper into biological tissues compared with the UV or VIS light required for the direct excitation of BSA–Au NC or Ce6 [33]. In addition, irradiation at 808 nm typically produces much lower photothermal effects than the commonly used 980 nm excitation for UCNPs, because water absorbs strongly at 980 nm [34]. Consequently, 808 nm irradiation reduces the risk of local heating and improves the safety profile of NIR-activated PDT, particularly in tissues with high water content [35].

The cells treated with the UCNP-BSA–Au nanoplatform followed by irradiation showed only a slight reduction in viability (89.6 ± 6.4%) and metabolic activity (95.7 ± 14.2%) (Figure 4E,G,H) compared to control cells, indicating the minimal cytotoxicity of the nanoplatform. In contrast, the cells incubated with the UCNP-BSA–Au-Ce6 nanoplatform and exposed to 808 nm irradiation exhibited a pronounced photodynamic effect. The number of viable cells decreased, while the number of propidium iodide-positive cells increased compared to non-irradiated controls (Figure 4C,F). Quantitative image analysis and a CCK-8 assay confirmed this effect, showing significant decreases in cell viability (47.0 ± 8.3%) and metabolic activity (29 ± 8.6%).

In general, in vitro PDT typically induces apoptosis as the predominant mechanism of cell death [36]. In our experiments, treatment with the UCNP-BSA–Au-Ce6 nanoplatform followed by irradiation resulted in clear morphological alterations and a higher number of propidium iodide-positive cells (Figure 4A–F). These observations indicate the disruption of plasma membrane integrity, suggesting that photodynamic treatment led to late apoptotic events or secondary necrosis [37]. Additionally, apoptosis-related cellular damage has also been reported in previous studies employing UCNP-Ce6 systems, which is consistent with the photodynamic effects observed in the present study [38,39,40].

Collectively, these results demonstrate that the UCNP-BSA–Au-Ce6 nanoplatform effectively mediates NIR-activated PDT. The absence of toxicity in control groups, combined with the strong loss of viability observed only after the irradiation of the Ce6-containing nanoplatform, supports the proposed energy transfer mechanism from UCNPs to Ce6, resulting in singlet oxygen generation and subsequent tumor cell death.

## 4. Conclusions

Combining multiple nanomaterials into a single multifunctional platform can expand their application potential and provide additional advantages. In this study, we described a UCNP-BSA–Au–Ce6 nanoplatform that integrates the key features of all three components. UCNPs enable deep tissue imaging and excitation under NIR irradiation; BSA–Au NCs provide colloidal stability, enhanced biocompatibility, and moderate ROS generation; and Ce6 ensures efficient singlet oxygen production. Specifically, we optimized the formation of the UCNP-BSA–Au–Ce6 nanoplatform and ensured its reproducibility. The high energy transfer efficiency between UCNPs and Ce6 enables effective ROS generation under NIR irradiation, as confirmed by in vitro experiments in breast cancer cells. In contrast, in the absence of irradiation, the UCNP-BSA–Au–Ce6 nanoplatform does not affect cell viability, enabling the precise control of the treatment area via light activation. Overall, we presented an NIR-activated, ROS-generating multifunctional nanoplatform that may advance cancer therapy research.

## Figures and Tables

**Table 1:** Efficiency of energy transfer from UCNP emission bands at 407 nm and 653 nm to Ce6.

UCNP Emission Band	UCNP:Ce6 Ratio						
	1:1	1:2	1:5	1:10	1:20	1:50	1:100
407 nm	0%	0%	3%	7%	9%	8%	9%
653 nm	9%	14%	22%	32%	31%	31%	29%

## Data Availability

The original contributions presented in this study are included in the article/Supplementary Material. Further inquiries can be directed to the corresponding author.

## Supplementary Materials

The following supporting information can be downloaded at https://www.mdpi.com/article/10.3390/nano16070417/s1, Figure S1: Zeta potential of BSA–Au NCs and ligand-free NaGdF_4_:Yb,Er@NaGdF_4_:Yb,Nd UCNPs in water as function of pH.; Figure S2: Photoluminescence and upconversion emission spectra of UCNP-BSA–Au nanoplatform before and after centrifugation; Figure S3: Spectral overlap between UCNP emission (energy donor) and the excitation spectrum of BSA–Au NCs as well as the absorption spectrum of chlorin e6; Figure S4. Photoluminescence decay kinetics of UCNPs and UCNP-BSA–Au and UCNP-BSA–Au–Ce6 nanoplatforms; Table S1: Parameters of emission decay kinetics of UCNPs and UCNP-BSA-Au and UCNP-BSA–Au–Ce6 nanoplatforms; Figure S5: Confocal microscopy images of UCNP-BSA-Alexa555 accumulation in MCF-7 cells; Figure S6: Viability of MDA-MB-231, MCF-7 and MCF-10A cells after 24 h and 48 h incubation with 0.1 mg/mL UCNPs and UCNP-BSA–Au and UCNP-BSA–Au-Ce6 nanoplatforms, without exposure to light. References [6,8,13,19,28,32,41] are cited in the Supplementary Materials.

## Abbreviations

The following abbreviations are used in this manuscript:

Au NCs	Gold nanoclusters
BSA	Bovine serum albumin
CCK-8	Cell counting kit-8
Ce6	Chlorin e6
DHR 123	Dihydrorhodamine 123
EP	Endoperoxide product
FRET	Förster resonance energy transfer
HSA	Human serum albumin
IEP	Isoelectric point
NIR	Near-infrared
PDT	Photodynamic therapy
PEG	Polyethylene glycol
PI	Propidium iodide
PL	Photoluminescence
PS	Photosensitizer
Rh123	Rhodamine 123
ROS	Reactive oxygen species
SOSG	Singlet Oxygen Sensor Green
SOSG-EP	Singlet Oxygen Sensor Green endoperoxide
UCNP	Upconverting nanoparticle
UV	Ultraviolet
VIS	Visible
