# Supplementary material for: Upconverting Nanoparticles Functionalized with Protein–Gold Nanoclusters and Chlorin e6 for Near-Infrared-Activated Photodynamic Therapy
Source: Nanomaterials (Basel). 2026 Mar 30;16(7):417. doi: 10.3390/nano16070417 (PMC13074957; doi:10.3390/nano16070417)
Supplement: Supplementary file 1 [file nanomaterials-16-00417-s001.zip › nanomaterials-4228452-supplementary.pdf]

Supplementary Materials

Upconverting Nanoparticles Functionalized with Protein–Gold Nanoclusters and Chlorin e6 for Near-Infrared-Activated Photodynamic Therapy

Vilius Poderys [1], Greta Butkiene [1], Dziugas Jurgutis [1], Aleja Marija Daugelaite [1,2], Egle Ezerskyte [1,2], Vaidas Klimkevicius [1,2] and Vitalijus Karabanovas [1,3,*]

1 Biomedical Physics Laboratory, National Cancer Institute, P. Baublio str. 3b, LT-08406 Vilnius, Lithuania
2 Institute of Chemistry, Faculty of Chemistry and Geosciences, Vilnius University, Naugarduko str. 24, LT-03225 Vilnius, Lithuania
3 Department of Chemistry and Bioengineering, Vilnius Gediminas Technical University, Sauletekio av. 11, LT-10221 Vilnius, Lithuania
* Correspondence: vitalijus.karabanovas@nvi.lt

Contents

1. Detailed Materials and Methods 2

1.1. BSA-Au NCs Synthesis 2

1.2. Core-shell UCNPs Synthesis 2

1.3. Formation of UCNP–BSA-Au–Ce6 Nanoplatform 3

1.4. Spectroscopic Measurements 4

1.5. Zeta Potential Measurements 4

1.6. Reactive Oxygen Spiecies Generation Measurements 4

1.7. Singlet Oxygen Generation Measurements 5

1.8. Cell Culturing 5

1.9. Cellular Uptake 5

1.10. In Vitro PDT Experiments 6

1.11. Evaluation of Cell Viability and Metabolic Activity 6

2. Supporting Results 7

2.1. Zeta Potential Measurements 7

2.2. UCNP-BSA-Au Nanoplatform Spectra Before and After Centrifugation 7

2.4. Decay Kinetics 8

2.5. Parameters of Emission Decays Kinetics 8

2.6. Accumulation of UCNP-BSA-Alexa555 in MCF-7 Cells 9

2.7. Cytotoxicity Evaluation of Nanoplatforms 9

3. References 10

1. Detailed Materials and Methods

1.1. BSA-Au NCs Synthesis

Gold nanoclusters stabilized with bovine serum albumin (BSA-Au NCs) were synthesized according to the protocol described by Xie et al. [1], with minor modifications. Briefly, a 9 mM aqueous $HAuCl_4$ solution was prepared and mixed with a 50 mg/mL bovine serum albumin (BSA) solution at 37 °C under vigorous magnetic stirring. Equal volumes (10 mL) of both solutions were combined, and after 2 min, 1 mL of 1 M NaOH was added to initiate reduction of Au^{3+} ions and formation of protein-stabilized nanoclusters. The reaction mixture was maintained at 37 °C for 16 h under continuous stirring. Following synthesis, purification and exchange of solvent was performed by dialysis using a 3.5 kDa molecular weight cut-off SnakeSkin™ membrane (Fisher Scientific, USA). Dialysis was carried out sequentially for 4 h, followed by replacement of the dialysate and an additional 4 h dialysis, and finally 16 h in fresh deionized water. The purified BSA-Au NCs solution was stored at 4 °C until further use.

1.2. Core-shell UCNPs Synthesis

The $NaGdF_4$:Yb^{3+}(18%),Er^{3+}(2%)@$NaGdF_4$:Yb^{3+}(5%),Nd^{3+}(40%) core-shell UCNPs were freshly synthesized before the experiments via a two-step thermal co-precipitation method followed by oleate ligand removal from the surface. UCNPs were obtained according to previously published procedures [2].

1.2.1. Synthesis of **$NaGdF_4$:18%Yb^{3+},2%Er^{3+}** Cores:

Stoichiometric amounts of freshly prepared aqueous solutions (0.2 M) of gadolinium(III) acetate ($Gd(OAc)_3$, 4 mL, 0.8 mmol), ytterbium(III) acetate ($Yb(OAc)_3$, 0.9 mL, 0.18 mmol), and erbium(III) acetate ($Er(OAc)_3$, 0.1 mL, 0.02 mmol) were poured into a 50 mL three-necked round-bottomed flask and dried to a solid at 90–95 °C. The flask was then cooled to room temperature, and a mixture of the dry acetates was dispersed in methanol (3 mL) under vigorous stirring. Subsequently, 10 mL of OA and 15 mL of ODE were added to the dispersion of the Ln-acetates, and the flask was placed in a heating mantle equipped with a PID temperature controller and a glass-coated thermocouple. The reaction mixture was kept under an Ar atmosphere and gradually heated to 120 °C to remove methanol and any traces of moisture. When the temperature reached 120 °C, the vacuum line was connected, and the reaction solution was maintained at 120 °C under reduced pressure (15 mbar) for 15 min, followed by raising the temperature to 140 °C. The vacuum line was then disconnected, and the flask was filled with Ar. Next, the temperature was increased to 150 °C and maintained for 40 min under the Ar atmosphere. After this step, the flask was cooled down to room temperature, and the prepared solutions of NaOH (1 M in MeOH, 2.5 mL, 2.5 mmol) and NH_4F (0.4 M in MeOH, 10 mL, 4 mmol) were mixed, shaken for 15 s, and poured into the reaction mixture at once. The obtained mixture was heated to 50 °C and maintained at this temperature for 30 min. Subsequently, the temperature gradually increased to 120 °C to remove methanol from the reaction mixture. When the temperature reached 120 °C, the vacuum line was connected, and the reaction solution was maintained at 120 °C under reduced pressure (15 mbar) for 30 min. The temperature was then increased to 310 °C and maintained for 1 hour (under an Ar atmosphere). Afterward, the reaction mixture was cooled to room temperature and poured into an excess of an acetone/hexane mixture (4:1 v/v, 150 mL). Pellets were collected by centrifugation at 10,000 rpm for 10 min, followed by four other washing steps: acetone, acetone/DI water mixture (1:1 v/v), acetone again, and finally with cyclohexane/acetone mixture (1:4 v/v). It should be noted that the pellets were collected by centrifugation (10,000 rpm, 10 min) after every wash. After the washing procedure was completed, the final pallets were redispersed in 20 mL of cyclohexane.

1.2.2. Formation of **$NaGdF_4$:5%Yb^{3+},40%Nd^{3+}** Outer Shell:

Stoichiometric amounts of freshly prepared aqueous solutions (0.2 M) of gadolinium(III) acetate ($Gd(OAc)_3$, 2.75 mL, 0.55 mmol), ytterbium(III) acetate ($Yb(OAc)_3$, 0.25 mL, 0.05 mmol), and neodymium(III) acetate ($Nd(OAc)_3$, 2 mL, 0.4 mmol) were poured into a 50 mL three-necked round-

bottomed flask and dried to a solid at 90–95 °C. The following synthesis steps are identical to those previously described (Synthesis of **$NaGdF_4$:18%Yb^{3+},2%Er^{3+}** cores). After 40 minutes at 150 °C, the reaction mixture was cooled down to room temperature, and 10 mL of the previously obtained core particles (**$NaGdF_4$:18%Yb^{3+},2%Er^{3+}**) was added to the mixture. The cyclohexane was removed using reduced pressure (at 120 °C), and the reaction mixture was cooled down to room temperature once again before adding the prepared solutions of NaOH (1 M in methanol, 2.5 mL, 2.5 mmol) and NH_4F (0.4 M in MeOH, 10 mL, 4 mmol). The remaining synthesis and purification procedures for obtaining the core–shell (**$NaGdF_4$:18%Yb^{3+},2%Er^{3+}@$NaGdF_4$:5%Yb^{3+},40%Nd^{3+}**) nanoparticles were identical to those of the core nanoparticles. After the washing procedure was completed, the final pallets were redispersed in 20 mL of cyclohexane. The UCNPs concentration was determined gravimetrically.

1.2.3. Oleate ligand removal procedure

5 mL of nanoparticle stock solution in cyclohexane was poured into a 50 mL centrifuge tube, mixed with 4-fold acetone (20 mL), and centrifuged at 12000 rpm for 15 minutes. The collected particles were mixed with deionized water (pH 3-4, adjusted with HCl) and vigorously stirred for 3 hours at room temperature. Subsequently, 10 mL of diethyl ether was added, and the aqueous/organic solution was mixed. The aqueous phase containing the oleate-free UCNPs was isolated using a separatory funnel. UCNPs were precipitated with acetone (1:3 v/v) and collected by centrifugation at 12000 rpm for 40 minutes. The UCNPs were redispersed in deionized water (pH 3-4), again precipitated with acetone (1:3 v/v), and collected via centrifugation. The UCNPs were then washed with 20 mL acetone and centrifuged again (12000 rpm, 40 minutes). Finally, the collected oleate-ligand-free UCNPs were redispersed in 10 mL of deionized water and stored at room temperature for further experiments. The concentration of the aqueous UCNPs dispersions was determined gravimetrically.

1.3. Formation of UCNP–BSA-Au–Ce6 Nanoplatform

UCNP–BSA-Au nanoplatform was prepared by mixing aqueous dispersions of UCNPs and BSA-Au NCs at defined molar ratios. For spectroscopic interaction studies, UCNPs concentration was adjusted to 1.43×10^{-7} mol/L (this corresponds to 0.5mg/mL), while BSA-Au NCs concentration was 100 times higher (1.43×10^{-5} mol/L)to ensure an excess of BSA–Au NCs, preventing them from becoming a limiting factor and allowing complete surface coverage of UCNPs by the nanoclusters.

To assess nanoplatform formation and surface association between UCNPs and BSA-Au NCs, emission spectra of freshly prepared mixtures were recorded prior to centrifugation. Samples were subsequently centrifuged at 14,500 RPM for 15 min (Eppendorf MiniSpin plus centrifuge, Eppendorf AG, Germany), and emission spectra of both supernatant and sediment fractions (redispersed pellet) were measured. A decrease in BSA-Au NCs related spectral features in the supernatant together with their presence in the sediment fraction was interpreted as evidence of nanocluster adsorption onto UCNP surfaces.

To enhance the photodynamic performance of the UCNP–BSA–Au NC system, chlorin e6 (Ce6) was incorporated into pre-formed UCNP–BSA–Au NC dispersions. The interaction between Ce6 and BSA-Au NCs was evaluated by comparing the fluorescence spectrum of free Ce6 in aqueous solution (1.43×10^{-7} mol/L) with that of a 1:1 mixture of BSA–Au NCs (1.43×10^{-7} mol/L) and Ce6 (1.43×10^{-7} mol/L). The observed bathochromic shift of the Ce6 fluorescence maximum in the presence of BSA–Au NCs indicated modifications in the local microenvironment of the photosensitizer upon association with the protein-containing complex.

Analogously, energy transfer processes within the ternary UCNP–BSA–Au–Ce6 system were evaluated by monitoring changes in UCNP emission decay kinetics. Measurements of bare UCNPs (1.43×10^{-7}), UCNP-BSA-Au nanoplatform (preparation described above) and UCNP-BSA-Au-Ce6 nanoplatform prepared using 1:1, 1:2, 1:10, 1:20, 1:50 and 1:100 UCNP:Ce6 ratios. These ratios correspond to Ce6 concentrations of 1.43×10^{-7}, 2.86×10^{-7}, 7.14×10^{-7}, 1.43×10^{-6}, 2.86×10^{-6}, 7.14×10^{-6} and 1.43×10^{-5} mol/L, respectively. The time-resolved photoluminescence measurements were performed at the UCNP emission bands in the blue (407 nm), green (549 nm), and red (653 nm) spectral

regions. UCNP emission lifetime shortening upon increasing Ce6 content was used as an indicator of non-radiative energy transfer. Energy transfer efficiency from UCNPs to Ce6 was determined using following equation [3]:

$$\eta = 1 - \frac{\tau_{UCNP-BSA-Au\,-Ce6}}{\tau_{UCNP}} \quad (1)$$

where η is the energy transfer efficiency, $\tau_{UCNP-BSA-Au-Ce6}$ is the UC emission decay lifetime of UCNP-BSA-Au-Ce6 nanoplatform, and τ_{UCNP} is the emission decay lifetime of the upconverting nanoparticles.

1.4. Spectroscopic Measurements

Steady-state emission spectra of UCNPs were measured using an FLS920 fluorescence spectrometer (Edinburgh Instruments Ltd., UK). Spectra were recorded using 1 nm emission slit width, 1 nm spectral step, and integration time of 0.1s using an R928 photomultiplier tube (PMT) detector. Measurements were carried out in quartz cuvettes (path length 10 mm) at 24 °C. Near-infrared laser emitting at 808 nm (Edinburgh Instruments Ltd., UK) with an excitation power of 1,9 W was used for excitation. Emission spectra were collected in the spectral range of 350 - 750 nm.

Time-resolved photoluminescence decay kinetics of UCNPs and UCNP-based nanoplatforms (UCNP–BSA–Au NC and UCNP–BSA–Au NC–Ce6) were measured using the FLS920 fluorimeter with R928 PMT detector operating in multichannel scaling (MSC) mode. The decay curves were recorded at the UCNP emission bands in blue (407 nm), green (549 nm), and red (653 nm) spectral regions. Emission slit for all time resolved measurements was set to 10 nm. An 808 nm laser operating in externally modulated pulsed mode was used for excitation. The repetition rate of the laser was 50 Hz with a pulse width of 16.6μs. The channel width of the detection system was 2 μs, decay curves were acquired over 2 ms time window. The decay curves were fitted using a bi-exponential decay model.

Absorption spectra of BSA–Au NCs were recorded using a Varian Cary 50 UV–Vis spectrophotometer (Varian Inc., Australia). Measurements were performed in quartz cuvettes (path length 10 mm) at room temperature.

Photoluminescence emission (λ_{ex}=405 nm) and photoluminescence excitation (λ_{em}=650 nm) spectra of BSA–Au NCs were recorded using a Cary Eclipse fluorescence spectrophotometer (Varian Inc., Australia). Measurements were performed with excitation and emission slit widths of 5 nm, and a scan rate of 240 nm/min. Excitation spectra were recorded in the range 350 - 580 nm, while emission spectra were recorded in the range 430 - 780 nm. Measurements were carried out in quartz cuvettes (path length 10 mm) at room temperature.

1.5. Zeta Potential Measurements

Zeta potential measurements were performed to evaluate the surface potential of UCNPs and BSA–Au NCs and to assess their colloidal stability. Measurements were performed in aqueous dispersions under various pH conditions. pH of solutions was adjusted by adding required amount of hydrochloric acid (HCl) or Sodium hydroxide (NaOH). Measurements of zeta potential were performed using Zetasizer Pro (Malvern Panalytical Ltd, UK).

1.6. Reactive Oxygen Spiecies Generation Measurements

UCNP–BSA–Au nanoplatform for ROS generation studies were prepared by mixing UCNPs with BSA–Au NCs at a molar ratio of 1:10, corresponding to UCNP and BSA–Au NC concentrations of 1.43 × 10^{-7} M and 1.43 × 10^{-6} M, respectively. ROS generation was evaluated by using dihydrorhodamine 123 (DHR123), a fluorescent probe sensitive to ROS. Dihydrorhodamine 123 (Invitrogen, USA) stock solution was prepared by diluting 6 μL of a 5 mM DHR123 solution with phosphate-buffered saline (PBS, pH 7.2) to obtain a 33 μM solution. The probe was added to the studied samples to achieve a final DHR123 concentration of 3.30 × 10^{-6} M.

Three types of samples were investigated: UCNPs, BSA–Au NCs, and UCNP–BSA–Au NC nanoplatform. An 808 nm diode laser (Edinburgh Instruments Ltd., UK) with an output power of 1.9 W was used for irradiation of samples. The laser beam diameter at the cuvette position was 1 mm.

Samples (0.7 mL) were placed in 10 mm path length semimicro cuvettes (Thermo Fisher Scientific, USA) and irradiated under constant magnetic stirring using a thermostated cuvette holder maintained at 24 °C. Because the samples were continuously stirred during irradiation, the entire solution volume was uniformly exposed to the incident radiation. Therefore, the irradiation dose presented in the graphs is expressed as energy per unit volume (J/cm^3) rather than energy per unit area (J/cm^2), which is typically used in photodynamic therapy experiments involving surface irradiation of biological tissues.

Fluorescence spectra were recorded after each irradiation interval using an FLS920 fluorescence spectrometer (Edinburgh Instruments Ltd., Livingston, UK). The excitation wavelength was 480 nm, and emission spectra were recorded in the 490–850 nm spectral range using an excitation slit width of 1 nm, emission slit width of 5 nm, spectral step of 1 nm, and dwell time of 0.1 s. All measurements were performed under identical irradiation and detection conditions to ensure comparability between different formulations. ROS generation was evaluated by changes of integrated the fluorescence intensity of the Rhodamine 123 emission band in the 515–550 nm spectral region.

1.7. *Singlet Oxygen Generation Measurements*

UCNP–BSA–Au–Ce6 nanoplatform for singlet oxygen generation studies was prepared by adding preformed BSA-Au NCs- Ce6 complex to the UCNPs solution thus forming BSA-Au NCs- Ce6 corona on UCNPs. Molar ratio of UCNP:Ce6 in prepared UCNP–BSA–Au–Ce6 nanoplatform was 1:4, resulting in final concentrations of 1.43×10^{-7} M, 1.43×10^{-6} M and 5.71×10^{-6} M for UCNP, BSA-Au NCs and Ce6 respectively. Ce6 was added in excess to ensure binding of the photosensitizer to all protein molecules forming the corona around the UCNPs. Singlet oxygen generation was evaluated using Singlet Oxygen Sensor Green (SOSG), a fluorescent probe selective for singlet oxygen. SOSG (Invitrogen, USA) stock solution was prepared by dissolving 100 μg of the probe in methanol (33 μL) and further diluting 12 μL of this solution with phosphate buffer (pH 7.4) to obtain a 50 μM SOSG stock solution. The probe was added to the studied samples to achieve a final SOSG concentration of 5×10^{-6} M.

Four types of samples were investigated: UCNPs, BSA–Au NCs, UCNP–BSA–Au NC nanoplatform, and UCNP–BSA–Au–Ce6 nanoplatform. An 808 nm diode laser (Edinburgh Instruments Ltd., UK) with an output power of 1.9 W was used for irradiation of samples. The laser beam diameter at the cuvette position was 1 mm. Samples (0.7 mL) were placed in 10 mm path length semimicro cuvettes (Thermo Fisher Scientific, USA) and irradiated under constant magnetic stirring using a thermostated cuvette holder maintained at 24 °C.

Fluorescence spectra were recorded after each irradiation interval using an FLS920 fluorescence spectrometer (Edinburgh Instruments Ltd., Livingston, UK). The excitation wavelength was 480 nm, and emission spectra were recorded in the 490–850 nm spectral range using an excitation slit width of 1 nm, emission slit width of 5 nm, spectral step of 1 nm, and dwell time of 0.1 s. All measurements were performed under identical irradiation and detection conditions to ensure comparability between different formulations. Singlet oxygen generation was evaluated by monitoring changes in the integrated fluorescence intensity of the SOSG-EP emission band in the 515–550 nm spectral region.

1.8. *Cell Culturing*

As a model *in vitro* system for cellular experiments, we have chosen two human breast cancer cell lines MDA-MB-231 and MCF-7 (purchased from the European Collection of Cell Cultures and American Type Culture Collection, respectively) and MCF-10A as a healthy breast tissue cell line (purchased from American Type Culture Collection). MDA-MB-231 and MCF-7 cells were cultured in a Dulbecco's Modified Eagle Medium (DMEM), supplemented with 10 % (v/v) fetal bovine serum (FBS), 100 U/mL penicillin and 100 μg/mL streptomycin (P/S) (all from Gibco, USA). MCF-10A cells were cultured in MEGM® Mammary Epithelial Cell Growth Medium BulletKit® (CC-3151, Lonza Bioscience, USA) supplemented with 100 ng/ml cholera toxin. All cells were maintained at 37 °C in a humidified atmosphere containing 5 % of CO_2. The cells were routinely subcultured 2–3 times a week in 25 cm^2 cell culture flasks.

1.9. *Cellular Uptake*

MBA-MB-231, MCF-7 and MCF-10A cells were seeded into an 8-well chamber slide with removable wells (Lab-Tek, Nunc, Thermo Fisher, Denmark) at a density of 15000 cells/well and maintained at 37 °C in a humidified atmosphere containing 5% of CO_2 for 24 hours. Cells were treated with 0.1 mg/mL of nanoparticles (UCNPs, UCNP-BSA-Au or UCNP-BSA-Au-Ce6) solution for 24 hours. After incubation, cells were fixed with 4% formaldehyde (Sigma-Aldrich, Germany) for 15 min. Cell nuclei were stained with 10 μg/mL of Hoechst 33342 (Thermo Fisher, USA), and actin filaments were stained with 5 U/mL Phalloidin-CF®594 conjugate (Biotium, Inc., US). The accumulation of UCNPs, UCNP-BSA-Au, and UCNP-BSA-Au-Ce6 was observed using a Nikon Eclipse Te2000-S C1 Plus laser scanning confocal microscope (Nikon, Japan) equipped with 405 nm (Melles Griot, USA), 543 nm (Melles Griot, USA) and 808 nm (BWT, China) continuous wave lasers. Imaging was performed using 60×/1.4 SA oil immersion objective (Nikon, Japan). The three-channel RGB detector filters (band-pass filters 450/17, 545/45 and 688/67 for blue, green and red channels, respectively) were used. Hoechst 33258 was excited at 404 nm, CF594 dye was excited at 543 nm, UCNPs were excited at 808 nm. Image processing was performed using the Nikon EZ-C1 Bronze version 3.80 and ImageJ 1.8.0_172 software.

1.10. In Vitro PDT Experiments

MDA-MB-231 cells were seeded into every second well of a 96-well plate (5×10^3 cells per well), leaving empty wells between experimental wells to prevent cross-irradiation. After 24 hours, cells were washed three times with Dulbecco's phosphate-buffered saline (DPBS; Gibco) containing Ca^{2+} and Mg^{2+}, without phenol red, and incubated with UCNP-BSA-Au or UCNP-BSA-Au-Ce6 nanoplatforms diluted in Opti-MEM (Gibco) supplemented with 100 U mL^{-1} penicillin and 100 μg mL^{-1} streptomycin for 24 hours. The final UCNP concentration in both groups was 0.1 mg/mL. Control wells were treated with deionized water added at the same final volume fraction (20% v/v) as used for the nanoplatform treated cells. Following incubation, cells were washed twice with DPBS and replenished with standard cell culture medium prior to irradiation.

Irradiation was performed using a custom-built system based on a modified Tevo Tornado 3D printer equipped with an 808 nm diode laser (K808DB2RN-4.000W, BWT Beijing Ltd., China) and a microplate holder. Individual wells of 96-well plates (Falcon) were irradiated with a 2 W laser beam, with a 1 mm beam diameter at the irradiation plane (irradiation area: 0.785 mm^2). The beam was scanned at 5mm/s across the entire well surface for 53 cycles in a line-scanning pattern with a step size of 0.5 mm between parallel lines. The total irradiation time per well was 945 s, corresponding to a total dose of 5000 J/ cm^2. After irradiation, cells were incubated for an additional 24 hours prior to viability assessment**.**

1.11. Evaluation of Cell Viability and Metabolic Activity

Cell viability was evaluated 24 hours after irradiation using 2 μM Calcein-AM (Invitrogen) for live-cell staining and 6 μM propidium iodide (PI; Carl ROTH, Karlsruhe, Germany) for dead-cell staining. After imaging, automated quantification of live and dead cells was performed using the ImageJ AutoCount macro [4], with parameters optimized to ensure accurate detection under varying cell confluency. The percentage of live cells was calculated as the ratio of Calcein-positive cells to the total number of Calcein and PI-stained cells.

Cell metabolic activity was assessed using the Cell Counting Kit-8 (CCK-8; Vazyme, China). At 24 hours post-irradiation, CCK-8 reagent was added to each well according to the manufacturer's instructions, and the plates were incubated for 2 hours under standard culture conditions. Absorbance at 450 nm was measured using a plate-reading spectrophotometer (BioTek Instruments, Thermo Fisher Scientific). Absorbance values were normalized to the experimental control group to determine relative cell metabolic activity.

Cytotoxicity of UCNPs and the nanoplatforms was tested on MDA-MB-231, MCF-7 and MCF-10A cells using the CyQUANT lactate dehydrogenase (LDH) assay (Invitrogen, Thermo Fisher Scientific, UK). Cells were seeded in 96-well plate (BD Falcon, USA) at 2×10^4 cells per well. After 24 hours, the cell

medium was replaced with medium containing 0.1 mg/mL UCNPs, UCNP-BSA-Au or UCNP-BSA-Au-Ce6. Cells were incubated with UCNPs or the nanoplatforms for 24 hours. Next day, an LDH assay was carried out according to the manufacturer's protocol. Absorbance at 490 nm and 630 nm was measured using a plate-reading spectrophotometer to quantify the concentration of extracellular LDH. Measured values were recalculated as viability of cells.

1.12. Statistical Analysis

All statistical analyses were conducted in R (version 4.4.3; R Core Team, 2025). Differences in viability or metabolic activity between control groups and the treated groups were evaluated using a two-sided Welch's t-test with Bonferroni correction. A significance level of α = 0.05 was used. Statistical significance within the graphs is indicated as one asterisk (*) for p-value < 0.05 and two asterisks (**) for p-value < 0.01.

2. Supporting Results

2.1. Zeta Potential Measurements

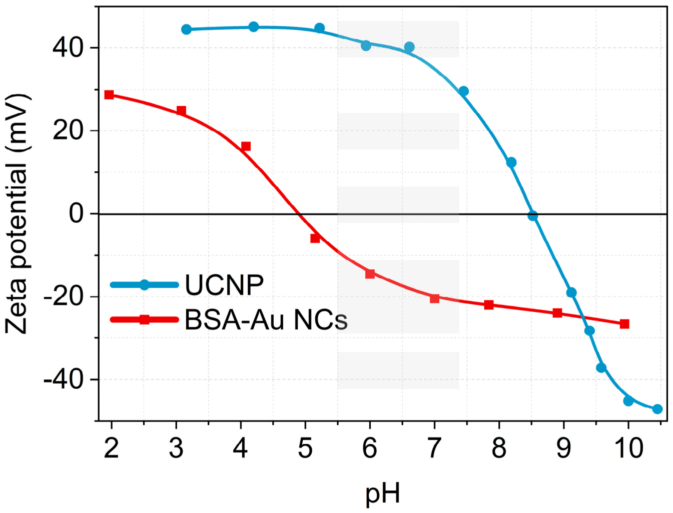

Figure S1. Zeta potential of BSA–Au NCs (red) and ligand-free $NaGdF_4$:Yb,Er@$NaGdF_4$:Yb,Nd UCNPs (blue) in water as a function of pH. The grey region indicates the pH range in which the formation of the BSA–Au NCs protein corona on UCNPs was performed.

2.2. UCNP-BSA-Au Nanoplatform Spectra Before and After Centrifugation

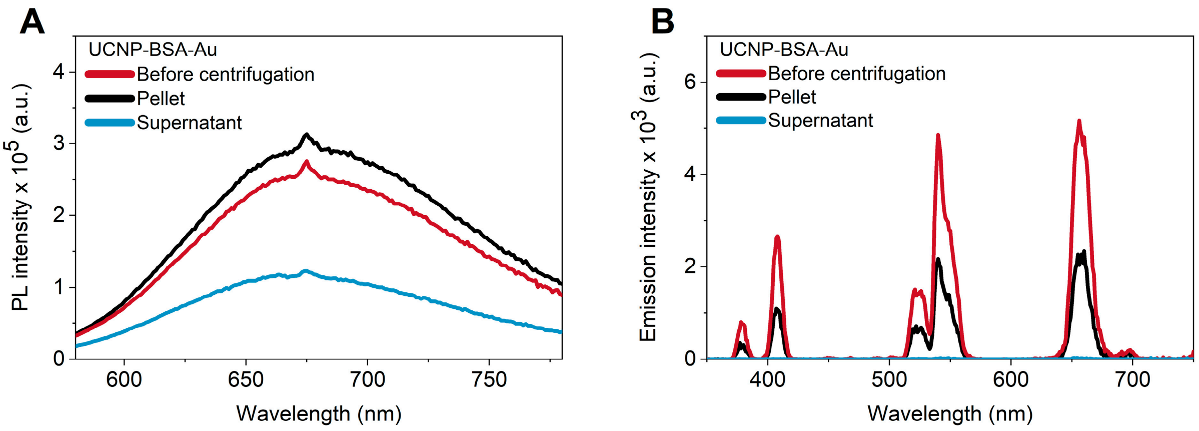

Figure S2. Photoluminescence and upconversion emission spectra of the UCNP–BSA–Au nanoplatform before and after centrifugation. (A) PL spectra (λ_{ex}=405nm) of the initial sample and the separated centrifugation fractions. (B) Corresponding upconversion emission spectra UCNPs-BSA-Au nanoplatform (λ_{ex}=808nm).

2.3. *Spectral Overlap of UCNP Emission, BSA–Au NCs Excitation and Ce6 Absorption Spectra*

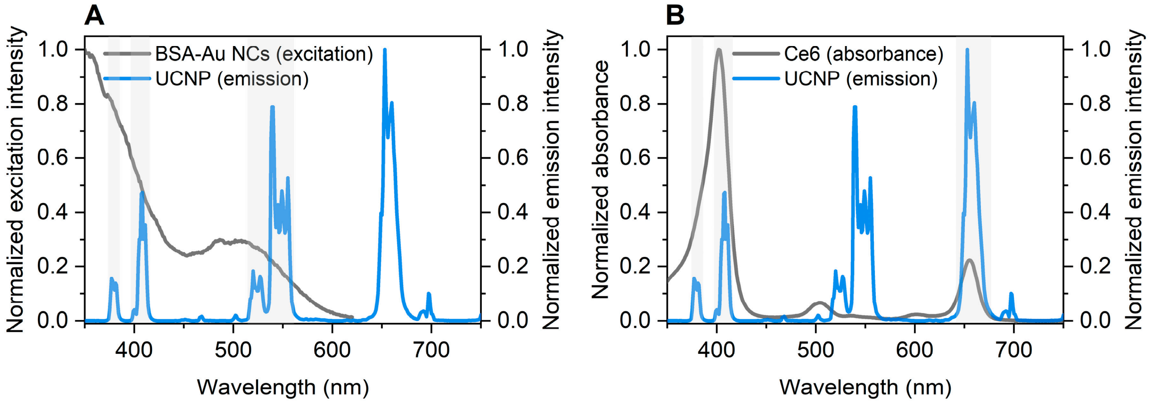

Figure S3. Spectral overlap between the UCNP emission (energy donor) and the excitation spectrum of BSA–Au NCs (A) as well as the absorption spectrum of chlorin e6 (B). The grey areas indicate the spectral regions where the overlap occurs, suggesting the possibility of energy transfer from UCNPs to the acceptor molecules.

2.4. *Decay Kinetics*

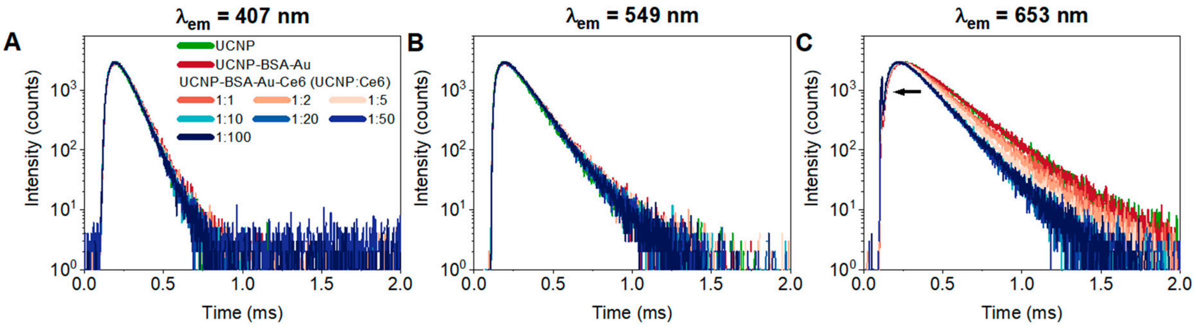

Figure S4. Photoluminescence decay kinetics of UCNPs, UCNP-BSA-Au and UCNP–BSA–Au–Ce6 nanoplatforms. (A) Decay kinetics of the 407 nm emission band. (B) Decay kinetics of the 549 nm emission band. (C) Decay kinetics of the 653 nm emission band.

2.5. *Parameters of Emission Decays Kinetics*

Table S1. Parameters of UCNPs, UCNP-BSA-Au and UCNP–BSA–Au–Ce6 nanoplatforms emission decays kinetics.

	407 nm			653 nm		
**Sample**	τ_1, µs	τ_2, µs	χ^2	τ_1, µs	τ_2, µs	χ^2
UCNP	29.9	74.1	0.660	49.5	212.6	1.493
UCNP-BSA–Au NC nanoplatform	34.3	76.0	0.620	50.6	213.2	1.553
UCNP-BSA–Au-Ce6 (1:1) nanoplatform	39.6	74.6	0.681	52.8	193.3	1.459
UCNP-BSA–Au-Ce6 (1:2) nanoplatform	33.4	73.9	0.909	52.8	182.1	1.489
UCNP-BSA–Au-Ce6 (1:5) nanoplatform	34.9	72.1	0.614	43.7	165.6	1.333
UCNP-BSA–Au-Ce6 (1:10) nanoplatform	38.4	68.7	0.715	45.0	145.0	1.159

UCNP-BSA–Au-Ce6 (1:20) nanoplatform	38.8	67.8	0.637	45.8	147.0	1.076
UCNP-BSA–Au-Ce6 (1:50) nanoplatform	38.1	68.3	1.214	48.4	147.0	1.181
UCNP-BSA–Au-Ce6 (1:100) nanoplatform	39.8	67.2	0.922	46.6	150.2	1.169

2.6. *Accumulation of UCNP-BSA-Alexa555 in MCF-7 Cells*

UCNPs were coated with BSA conjugated to Alexa555 dye (BSA-Alexa555), to evaluate if BSA coating remains attached to the UCNPs after internalization in cells. The overlapping UCNPs emission and BSA-Alexa555 fluorescence signals indicate that UCNP-BSA-Alexa555 nanoplatform remains stable after internalization (Figure S4).

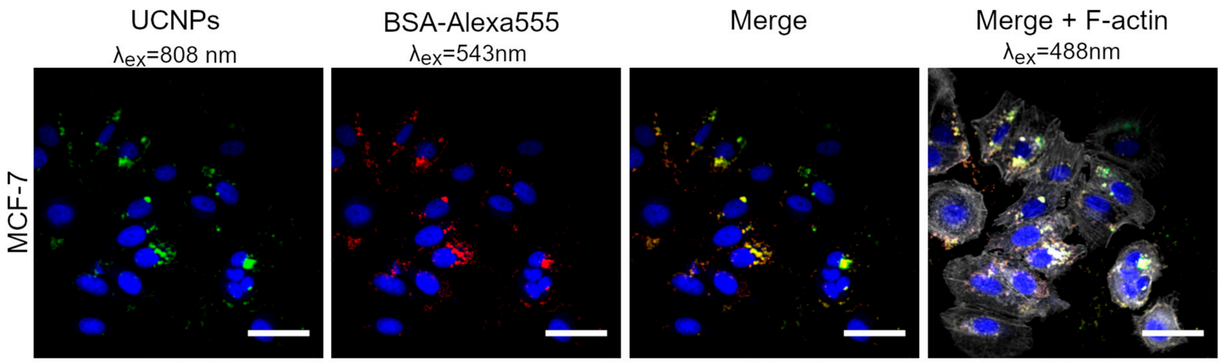

Figure S5. Confocal microscopy images of UCNP-BSA-Alexa555 accumulation in MCF-7 cells. Emission of UCNPs is represented as green (λ_{ex}=808 nm), fluorescence of Alexa555 dye is represented as red (λ_{ex}=543 nm). Nuclei of cells were stained with Hoechst (blue color, λ_{ex}=404 nm), F-actin was stained with Phalloidin-Alexa488 (white color, λ_{ex}=488 nm). Scale bars: 50 μm.

2.7. *Cytotoxicity Evaluation of Nanoplatforms*

Lactate dehydrogenase release assay was performed to evaluate whether UCNPs, UCNP-BSA-Au and UCNP-BSA-Au-Ce6 nanoplatforms are biocompatible and do not cause cytotoxicity without irradiation with light (in the dark). The assay results, shown in Figure S6, demonstrate that UCNPs, UCNP-BSA-Au and UCNP-BSA-Au-Ce6 nanoplatforms are biocompatible, when incubated in dark. This was expected since all components of nanoplatforms are non-toxic without light activation [5–7].

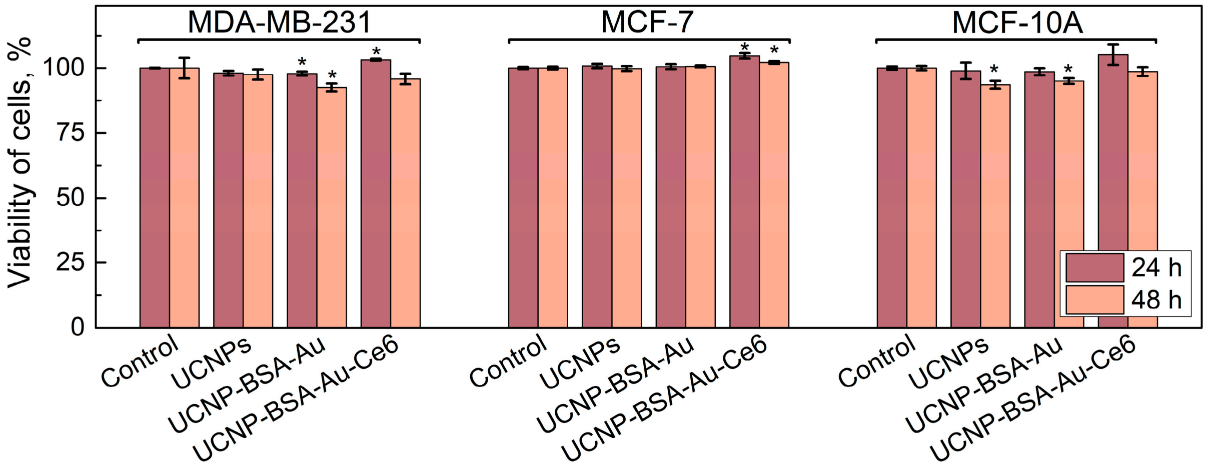

Figure S6. Viability of MDA-MB-231, MCF-7 and MCF-10A cells after 24 h and 48 h incubation with 0.1 mg/mL UCNPs, UCNP-BSA-Au and UCNP-BSA-Au-Ce6 nanoplatforms, without exposure to light. * represents statistically significant difference compared to control cells ($p<0.05$).

3. References

1. Xie, J.; Zheng, Y.; Ying, J.Y. Protein-Directed Synthesis of Highly Fluorescent Gold Nanoclusters. *J Am Chem Soc* **2009**, *131*, 888–889, doi:10.1021/ja806804u.
2. Ezerskyte, E.; Morkvenas, A.; Venius, J.; Sakirzanovas, S.; Karabanovas, V.; Katelnikovas, A.; Klimkevicius, V. Biocompatible Upconverting Nanoprobes for Dual-Modal Imaging and Temperature Sensing. *ACS Appl. Nano Mater.* **2024**, *7*, 6185–6195, doi:10.1021/acsanm.3c06111.
3. Dukhno, O.; Przybilla, F.; Collot, M.; Klymchenko, A.; Pivovarenko, V.; Buchner, M.; Muhr, V.; Hirsch, T.; Mély, Y. Quantitative Assessment of Energy Transfer in Upconverting Nanoparticles Grafted with Organic Dyes. *Nanoscale* **2017**, *9*, 11994–12004, doi:10.1039/c6nr09706e.
4. Sharara, A.; Kraft, C.; Shameem, M.; Singh, B.N. AutoCount: An ImageJ Macro for Automatic Cell Counting of Fluorescent Images. *DNA and Cell Biology Reports* **2025**, *6*, dcbr.2025.0032, doi:10.1089/dcbr.2025.0032.
5. Matulionyte, M.; Dapkute, D.; Budenaite, L.; Jarockyte, G.; Rotomskis, R. Photoluminescent Gold Nanoclusters in Cancer Cells: Cellular Uptake, Toxicity, and Generation of Reactive Oxygen Species. *Int J Mol Sci* **2017**, *18*, E378, doi:10.3390/ijms18020378.
6. Poderys, V.; Jarockyte, G.; Bagdonas, S.; Karabanovas, V.; Rotomskis, R. Protein-Stabilized Gold Nanoclusters for PDT: ROS and Singlet Oxygen Generation. *Journal of Photochemistry and Photobiology B: Biology* **2020**, *204*, 111802, doi:10.1016/j.jphotobiol.2020.111802.
7. Ezerskyte, E.; Butkiene, G.; Katelnikovas, A.; Klimkevicius, V. Development of Biocompatible, UV and NIR Excitable Nanoparticles with Multiwavelength Emission and Enhanced Colloidal Stability. *ACS Mater. Au* **2025**, doi:10.1021/acsmaterialsau.4c00151.
